# Keep on running – a randomized controlled trial to test a digital evidence-based intervention for sustained adoption of recreational running: rationale, design and pilot feasibility study

**DOI:** 10.1080/21642850.2021.1885410

**Published:** 2021-03-01

**Authors:** Hugo V. Pereira, Pedro J. Teixeira, Marta M. Marques, Eliana V. Carraça, Marlene N. Silva, Jorge Encantado, Inês Santos, António L. Palmeira

**Affiliations:** a Centro Interdisciplinar para o Estudo da Performance Humana (CIPER), Faculdade de Motricidade Humana, Universidade de Lisboa, Cruz Quebrada-Dafundo, Portugal; b Centro de Investigação em Desporto, Educação Física, Exercício e Saúde (CIDEFES), Universidade Lusófona, Lisboa, Portugal; c ADAPT SFI Research Centre and Trinity Centre for Practice & Health Care Innovation, Trinity College Dublin, Dublin, Ireland; d ISPA Instituto Universitário, APPsyCI – Applied Psychology Research Center Capabilities & Inclusion, Lisboa, Portugal; e Laboratório de Nutrição, Faculdade de Medicina, Universidade de Lisboa, Lisboa, Portugal

**Keywords:** Sports and exercise, motivation, self-regulation, behavior, web-based

## Abstract

**Background:**

This paper describes the rationale, intervention development, study design and results from the pilot feasibility study of the Keep On Running (KOR) trial. KOR aims to test a web-based brief theory-based intervention, targeting maintenance of recreational running behavior over time (i.e. relapse preventing).

**Methods:**

Intervention development was based both on Self-Determination Theory and on Self-Regulation Theory. As part of it, a pilot study was implemented (*n*=18) to measure intervention adherence and participant satisfaction in order to establish the feasibility and acceptability of the intervention toolkit. Furthermore, this pilot study was also used to test the feasibility and acceptability of the questionnaires selected to be part of the later RCT.

**Results:**

Pilot intervention acceptability was good, but overall adherence was low. Features such as feedback and social sharing should be added to the toolkit. The main trial should lessen questionnaire length and include data from usual monitoring gadgets and apps (APIs). The protocol of the RCT was adjusted to test the efficacy of the refined final version of the intervention, and the RCT that will test it, contributing to the understanding of recreational running sustainability, allowing the optimization of future interventions aimed at physical activity promotion.

## Background

While benefits of regular physical activity (PA) and exercise are well established (Kraus et al., 2019; Oja et al., 2015), insufficient PA and the prevalence of sedentary behavior remain as challenges in health promotion (Ding et al., 2016; Guthold, Stevens, Riley, & Bull, 2018). Although research suggests that behavior change interventions can increase PA over the course of an intervention, these effects are generally not persistent after the intervention ends (McEwan, Rhodes, & Beauchamp, 2020).

Along with initial involvement, sustained adherence is a well-known challenge in exercise contexts, as many people struggle to keep up their behavior for longer than 6 months (Kahlert, 2015; Marcus et al., 2000). Research on the characterization of people who are physically active (Cortis et al., 2017; Sawyer, Ucci, Jones, Smith, & Fisher, 2017) and manage to sustain their PA behavior (Amireault, Godinb, & Vézina-Imb, 2013) is extensive. While most of the research about behavior sustainability report how many discontinue practice, only few studies addressed the psychological mechanisms of behavior maintenance (Kwasnicka, Dombrowski, White, & Sniehotta, 2016), such as the quality of their motivation, the emotions and gains they experience while exercising, or the regulatory resources they use to overcome exercise-related challenges and barriers.

Self-determination theory (SDT) (Deci & Ryan, 2000, 2008) can provide a valid framework to study PA maintenance. The underlying premise of SDT is that sustained motivation is elicited from within the person (not imposed by someone else) and that supporting clients’ basic needs for autonomy (need to feel a sense of choice, volition and self-endorsement), competence (need to feel a sense of mastery and capacity to accomplish the behavior), and relatedness (need to feel meaningfully connected to others, valued and understood) will best promote the initiation and maintenance of behavior change, by fostering autonomous motivation and well-being. Conversely, when these three needs are thwarted, people will tend to develop controlled motivations, regulating their behavior based on external contingencies and internalized self-judgments (Vansteenkiste & Ryan, 2013).

In addition, not all types of goals have the same consequences (Ryan, Sheldon, Kasser, & Deci, 1996). The outcomes that individuals are pursuing through the new behavior can have intrinsic or extrinsic qualities, which can also influence behavior maintenance. Relative to ‘extrinsic goals’ (e.g. wealth, social recognition, physical attractiveness), ‘intrinsic’ goals (e.g. health, personal growth, social connectedness) tend to be regulated by more self-determined forms of behavioral regulation and are thought to result in improved self-regulation and longer-term outcomes (Ingledew & Markland, 2009).

In summary, SDT interventions have a significant effect on health behavior change (Sheeran et al., 2020). Additionally, SDT-informed interventions positively affect indices of health, partly due to increases in internalization, self-determined motivation and support from social agents, and were stronger at the end of interventions than at follow up (Ntoumanis et al., 2020). The effect sizes seen in PA interventions are mediated by our current theories (Rhodes, Boudreau, Josefsson, & Ivarsson, 2020). Taken together, recent evidence suggests that SDT (Deci & Ryan, 2000) can provide a valid framework to study PA maintenance. Furthermore, aligning motivational and post-motivational (i.e. self-regulatory) features represents a promising avenue for lasting behavior change (Hagger & Chatzisarantis, 2014). In this regard, self-regultory skills such as self-monitoring, individualized goal setting or action planning, have been identified as important mediators of the effect of interventions on long-term physical activity and as potentially core features of effective behavior change/maintenance interventions (Rhodes et al., 2020).

Thousands of health-related applications (apps) are available world-wide for smartphones and represent a unique opportunity to reach a broad audience of users. The most popular apps are for exercise, diet, and weight management, and 500 million users use mobile health applications. Mobile health interventions have surged in popularity but their implementation varies widely, and evidence of effectiveness is mixed (Dugas, Gao, & Agarwal, 2020). When trying to understand design features through the SDT lens, only one-fourth of the sample provided users support for the three basic psychological needs of competence, autonomy and relatedness (Villalobos-Zúñiga, 2020). It was found that prompts and cues, techniques of personalization, feedback and monitoring, goal setting and action planning were most commonly used in effective mobile health interventions (Dugas et al., 2020).

### The case for recreational running

Running is one of the most popular forms of leisure-time exercise (Andersen, 2020; Running-USA, 2020; Teixeira, Marques, Lopes, Sardinha, & Mota, 2019) in part because it is inexpensive and can be performed anywhere, almost at any time. In addition, it requires little technical skills, is relatively safe, and it is easy to learn. The health benefits of running are vast, including prevention of obesity, hypertension, dyslipidemia, type 2 diabetes, osteoarthritis and hip replacement, benign prostatic hypertrophy, respiratory disease, cancer, disability, reduction of cardiovascular, and all-cause mortality (Lavie et al., 2015; Lee et al., 2014; Pedisic et al., 2019).

Running stimulates the interest of the research community, especially because of the long hours of training throughout the year and the large number of events runners participate in (Zach et al., 2017). This suggests that motivational aspects related with training for and completing a race may be unique (Zach et al., 2017). With this in mind, running clubs/groups may provide a novel testbed for understanding why people adopt recreational running and how motivational factors can support PA maintenance.

To better promote running sustainability and its long-term health benefits, it is crucial to understand individuals’ running experiences and outcomes, as well as the factors that predispose them to engage in this activity. Studying the antecedents and outcomes of running motivation, as well as the efficacy of different types of interventions to promote running behavior will enhance the understanding of the phenomenon, possibly creating new insights into the effective promotion of PA maintenance.

To our knowledge, no previous study has been designed to test the efficacy of interventions to promote sustained running behavior and identify the mechanisms behind it. Future interventions will aim to test if motivation and self-regulation mediators can be successfully modified via a digital-based intervention. This will ultimately lead to sustained adherence to an activity that participants have chosen and appear to enjoy, via large-reach interventions, capitalizing on the widespread availability of running clubs/events and increased reliance of exercisers on remote technologies.

This paper describes the rationale, intervention development, study design, and pilot study of the Keep On Running (KOR) trial. KOR aims to test a brief theory-based intervention, delivered through digital technology (web app), targeting maintenance of recreational running behavior over time (i.e. relapse prevention) after being voluntarily initiated in the previous 3 months. The pilot study was focused on intervention adherence and participant satisfaction in order to establish the feasibility and acceptability of the intervention toolkit. Furthermore, it was also used to test the feasibility and acceptability of the questionnaires selected to be part of the later RCT.

### Study objectives and hypotheses

The study's objective is to develop and present a new digital toolkit aiming at maintenance of recreational running behavior over time (i.e. relapse preventing), and evaluate whether it can be delivered and accepted by participants. We hypothesize that the built intervention can be delivered and will be accepted by the participants, and tested by the RCT.

## Methods

### Study design

**Figure 1.**
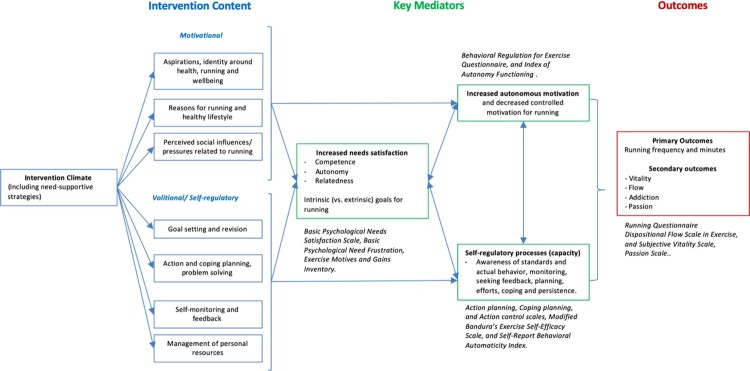
KoR's Logic Model.

KOR is a Randomized controlled trial (RCT). It was developed based on a logic model (Figure 1), adapting the motivational and self-regulation arm of the NoHoW study (Marques et al., 2020; Scott et al., 2019). The trial involved combinations of two conditions: (a) organized (i.e. Programa Nacional de Marcha e Corrida – PNMC) vs. free recreational runners (FRR), and (b) self-regulation and motivation (SRM) intervention vs. no intervention.

**Figure 2.**
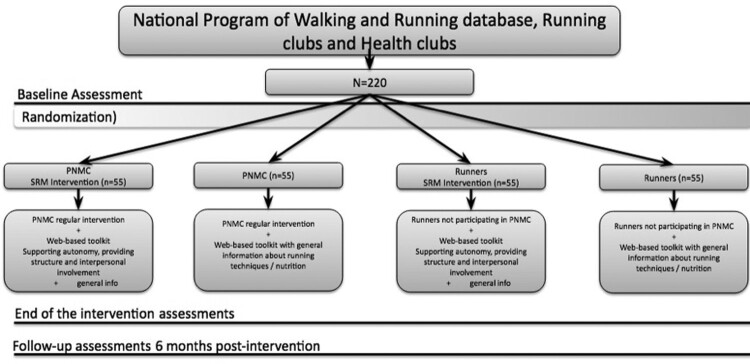
Organization of participant groups.

As a result, it comprises four different groups (Figure 2): (i) a group of runners enrolled in the running program and additionally receiving the theory-based intervention (PNMC + SRM); (ii) a group of runners merely participating in the organized running program (PNMC); (iii) a group of FRR receiving the theory-based intervention (FRR + SRM); and lastly, (iv) a group of FRR receiving only general information on running (i.e. control group).

**Figure 3.**
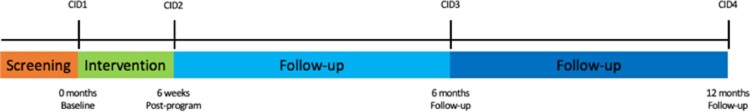
Participants’ timeline (CID – clinical investigation day).

This approach was taken because (a) comparing runners belonging to PNMC with FRR (i.e. control group) allows examining the effect of the PNMC on behavior maintenance; (b) comparing FRR enrolled in the SRM intervention with FRR allows testing the effectiveness of a light-touch intervention on motivational and other psychological variables, and on behavior maintenance; and (c) comparing runners belonging to PNMC and receiving the SRM intervention with FRR receiving the SRM intervention will allows examining whether the SRM intervention has an additional effect on the study outcomes. Trial duration is one year with assessments at 6 weeks, and 6 and 12 months postbaseline (Figure 3).

### Eligibility criteria

*Inclusion criteria*: age between 18 and 65 years; running for no longer than 3 months; performing a minimum of 60 min of vigorous physical activity per week; being free from major orthopedic limitations (defined as those which would limit moderate to vigorous physical activity (MVPA)); and from clinical diagnosis with any condition that may interfere with performing MVPA; being free from untreated or major psychological disorders (e.g. psychosis, clinical depression); and possessing or willing to acquire a smartphone or computer with internet access.

*Exclusion criteria*: inability to provide written informed consent; inability to follow written material or telephone conversations in Portuguese that would preclude completion of study questionnaires and use of the digital intervention (i.e. KOR toolkit, see below); intention of pregnancy in the next 12 months; planning to travel for more than 2 weeks in the next 12 months or to emigrate; suffering any injury in the course of the study.

### Motivation and self-regulation intervention

We developed a web-based intervention adapted from the motivational and self-regulation arm of the NoHoW tria (Marques et al., 2020; Scott et al., 2019). It consists of 11 short sessions divided in 8 modules with motivational and self-regulation based exercises, quizzes, videos and animations aimed to elicit reflection about motives, goals, barriers and strategies to foster running behavior sustainability. Beyond the long-term promotion of running behavior, by implementing theory-based and evidence-based behavior change techniques, this brief intervention aims at promoting participants’ intrinsic and well-internalized motivations for running and the use of self-regulation skills (e.g. goal setting) that facilitate behavior maintenance.

**Figure 4.**
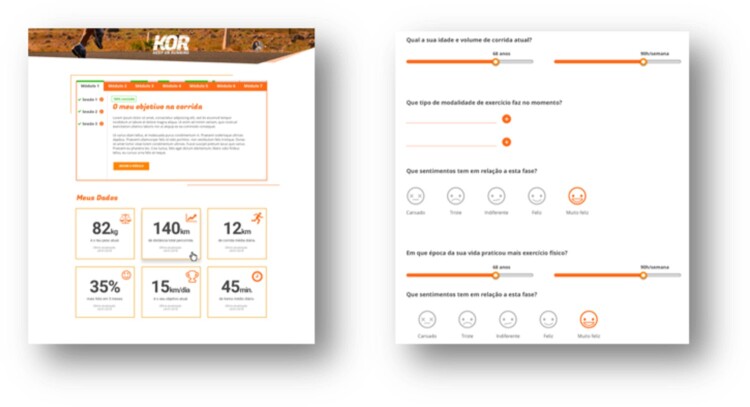
Screenshots of the intervention toolkit.

The intervention includes:

- Access to a web-based intervention comprising 11 distinct sessions of 5–10 min each and 8 educational modules concerning motivation and self-regulation constructs, delivered through several technological implementations, including videos, animations, quizzes and exercises, as well as images, text and audio (Figure 4). Several multimedia tools, including a web-based HTML5 interactive map as a navigation tool for the SRM sessions (http://leafletjs.com/), the questionnaires platform to design interactive features, and whiteboard drawings to convey some of the more theoretical constructs of the intervention (http://www.videoscribe.co/), were used to develop these implementations, supported by the team's previous experience in similar interventions.
- Bi-weekly text messages (see Table 1 for an overview of weekly text messages to participants) are sent to prompt participants toward the sessions and each week specific contents. Yet, participants are able to move faster or slower through the program to fit their needs and availability.

**Table 1.** Overview of weekly text messages to participants.

Text reminder 1	Good afternoon runner, you have already registered on the website http://kor.fmh.ulisboa.pt/. By now, you will have received an email to validate your account. Good races!
Text reminder 2	Good morning Athlete, have you had the opportunity to explore the contents and activities of the KoR program? Good Sunday wishes.
Text reminder 3	Good morning Athlete, how about a visit to the KoR program during today? This is the third of 11 reminders. Good races
Text reminder 4	Good morning Athlete, how is the exploitation of KoR content RUNNING? Good races
Text reminder 5	Good afternoon Athlete, you can do the activities of the KoR program at your own pace. How's your run going? Good races
Text reminder 6	Good afternoon Athlete, fortunately tomorrow is already cooling. We have now spent half of the KoR program. We hope you’re enjoying it. Good runs (in the shade)
Text reminder 7	Good afternoon, this is the 7th of 11 messages. At this point, you may have finished exploring the content or just be at the beginning. You can continue to enjoy content at your own pace. Good races
Text reminder 8	Good morning, the heat returned and we are on the final stretch of the KoR program. On vacation or at work we hope you are enjoying the program and your races. Good races
Text reminder 9	Good afternoon athlete! It is now just a little bit to finish your trip in the KoR toolkit. I hope you’re enjoying it. Good races
Text reminder 10	Good morning athlete! Weekend it is here, and a nice time for a run. This is the penultimate sms of the KoR program. You have certainly had the opportunity to explore the contents of the toolkit and reflect on your run. Best wishes for a great weekend
Text reminder 11	Good afternoon athlete! This will be the last message / reminder to access the KoR program. During this week, you will also be able to access the contents and register your race. Soon I will send you the link to complete the completion questionnaire. Have a nice week and good races

The SRM intervention includes both motivation and self-regulation components. The intervention's theoretical logic model is adapted from the NoHoW project (Marques et al., 2020; Scott et al., 2019) and presented in Figure 1. The self-regulation skills component will seek to empower users to identify goals and make action plans for when, where, and how the goals will be implemented, allowing them to formulate explicit implementation intentions. Coping plans for relapses will also be supported. The motivational component is therefore based on SDT and configured to optimally support participants’ psychological needs for autonomy, competence, and relatedness, via specific technological implementations (e.g. self-awareness exercises, videos and practice modules), and as a result improve autonomous regulation of physical activity behaviors. Participants will explore personal motives to run, skills, life goals, social connections, and other motivational elements. Messages, videos, animations, self-awareness questionnaires, among other tools, will stimulate users’ sense of volition and ownership (vs. external pressure), confidence and competence (vs. unpreparedness and sense of failure), and positive social support (vs. isolation). It is anticipated that the use of these implementations will increase autonomous self-regulation and intrinsic motivation, which are associated with longer-term behavior change, and positive health outcomes.

The SRM web-based toolkit will be accessible only to the groups receiving the SRM intervention. In addition, all the study participants will receive weekly email messages with general technical information about running. An individual telephone session will introduce runners to specific elements of their unique intervention and how it will unfold.

### Recruitment and randomization

Part of the runners will be recruited from the PNMC program, which consists in 2–3 supervised running group-sessions of 10–20 runners per week, lasting 90 min each. Certified trainers lead these sessions, apply specific methodologies for running and for walking sessions. Prior to their enrollment, trainers must complete a training course. The other set of runners (which run freely, on their own) will be recruited from the community (recreational runners). To recruit this sample, we will use online advertisement and running events list servers, reach out for running clubs, and partnerships with both state and private organizations.

We will use a rolling recruitment strategy: participants will join the study as soon as they are considered eligible to facilitate management of available human resources. Each set of runners will be randomly assigned to the KoR intervention group or the control group general information group in a 1:1 ratio, stratified by group (PNMC and FRR). The method of randomized permuted blocks will be used, with random block lengths (4 or 6) (Broglio, 2018). Because participants will know which arm of the study they are in, blinding is not possible.

### Assessments

Data on demographics (age, gender, date of birth, education, income, employment, marital status, weight, height, physical activity history, and personal characteristics) and potential psychological moderators (i.e. index of autonomous functioning) will be collected only at baseline.

The primary outcome of this study is running behavior, which will be measured at baseline, intervention's end and 6-month follow-up by self-report (frequency and minutes of running per week). At 12 months, participants will be asked by telephone whether they keep running (at least) at their initial level, or not.

Secondary outcomes will include putative mediators and outcomes. Basic psychologic need satisfaction will be assessed via the Psychological Need Satisfaction in Exercise Scale (Wilson, Rogers, Rodgers, & Wild, 2006); Basic psychological need frustration will be assessed trough the Basic Psychological Need Frustration in Exercise Scale (Chen et al., 2015); Motives and gains via Exercise Motives and Gains Inventory (Ingledew, Markland, & Strömmer, 2014); Behavioral regulations will be assessed via the Behavioral Regulation in Exercise Questionnaire – 3 (Cid et al., 2018); Self-determination trait will be assessed via the Index of Autonomy Functioning (Weinstein, Przybylski, & Ryan, 2012); Self-regulation skills will be measured trough the Action planning, Coping planning, and Action control scales (Sniehotta, Scholz, & Schwarzer, 2005a; Sniehotta, Schwarzer, Scholz, & Schuz, 2005b); Self-efficacy will be assessed via the Modified Bandura's Exercise Self-Efficacy Scale (Bandura, 1997); Automaticity will be measured trough the Self-Report Behavioural Automaticity Index (Gardner & Tang, 2014; Gardner, Abraham, Lally, & de Bruijn, 2012); and exercise identity via the Exercise Identity Scale (Anderson & Cychosz, 1994). Regarding psychological outcomes, flow will be measured through the Dispositional Flow Scale – 2 in Exercise (Jackson & Eklund, 2002); Vitality will be assessed through the Subjective Vitality Scale (Ryan & Frederick, 1997); and passion through the Passion Scale (Cid et al., 2019).

### Data collection

Participants will go through four assessment periods at baseline, 6 weeks (post-program), 6 months, and 12 months follow-up. The training and clarification session will be completed by telephone to ensure participants are equipped with the skills to access the intervention contents. Before the first, third and fourth assessment periods, eligibility will be checked against inclusion and exclusion criteria through a telephone screening/re-screening call, 1 week prior to each visit. Participant's timeline is shown in Figure 3.

*Assessment* 1 (baseline): Clarification session about the study. If participants are still interested in enrolling in the study, eligibility will be checked, and they will be asked to fill out an informed consent form. Randomization and allocation to one of the four arms of the trial will be performed. Data on demographics and potential psychological moderators will be collected. A battery of psychological and behavioral questionnaires will be completed online using google forms (in one-week time) – running behavior (i.e. frequency and minutes per week), motivations, basic psychological needs, motives and gains, self-regulation skills, passion, flow, and vitality. This task is estimated to take approximately 1 h, but participants will be able to do breaks and return later to complete the psychometric evaluation during that week. Debrief on how to use the KOR web-based intervention content – the toolkit (approximately 1 h).

*Assessment*s 2 and 3 (intervention's end and 6-month follow-up). Email reminder prior to this evaluation period will be sent. The same battery of psychological and behavioral questionnaires will be completed online using google forms (in one-week time) – running behavior (i.e. frequency and minutes per week), motivations, basic psychological needs, motives and gains, self-regulation skills, passion, flow, and vitality –, and will take approximately 1 h.

*Assessment* 4 (12-month follow-up). Running behavior maintenance will be assessed through a telephone call, asking participants whether they keep running at their initial level or higher, or not.

### Analytical strategy

Statistical analysis will be performed using SPSS Statistics version 25.0 (SPSS Inc., an IBM Company, Chicago IL, USA). Descriptive statistics will be calculated (mean, standard deviation and range). Repeated measures with Bonferroni corrections for adjusted comparisons will be used to examine differences between each intervention group(PNMC or SRM) and controls (i.e. free recreational runners), at all assessment points, in the primary and secondary outcomes measured. Statistical significance will be set at *p* < 0.05.

Mediation analysis, will test the psychological mechanisms underlying changes in running behavior from baseline to post program, 6 and 12 months. Multilevel modeling will be used to determine the SRM intervention effect on these motivational variables, with intervention group (SRM vs. Control group) as the between-subject factor and change in self-regulation and competence for running at baseline, 6–8 weeks, 6months and 12 months, serving as dependent variables. If necessary, multiple imputation methods will be implemented to provide robust results for primary and main secondary outcomes.

### Power calculations and sample size estimation

Two types of sample size calculations were made: (a) using the primary outcome – running minutes and (b) using secondary outcomes, in particular, changes on key psychosocial variables. Regarding our primary outcome, we have used vigorous physical activity for our calculations. A small effect size (Cohen's *d *= 0.23) is expected, a value previously registered in studies with intrinsic motivation interventions (Ntoumanis et al., 2020; Sheeran et al., 2020). Considering a power of 0.8 with two-tailed analysis, we estimated that each intervention group should have 37 (*p *= 0.05) or 55 participants (*p *= 0.01). Concerning our secondary outcomes, we are expecting effects sizes ranging from 0.23 to 0.73, based on previous reports (Ntoumanis et al., 2020; Sheeran et al., 2020). Thus, assuming an effect size of 0.50, 18 subjects/group will be required to detect significant differences at *p *= 0.05 and 28 for *p *= 0.01. Given that the primary outcome is expected to have a smaller effect size, and expecting a dropout rate of 15% at follow-up, we estimate that approximately 55 subjects/group are needed at baseline (for *p *= 0.05). Thus, recruiting 220 participants will ensure high statistical power for our primary analyses, extending its power to the secondary analyses.

Trial results will be published in peer-reviewed scientific journals and in several international and national scientific conferences. The website will be our central media to promote the dissemination of the results to the general public. Other media will be reached through university partners and website.

### Pilot feasibility study

This pilot study is focused on intervention adherence and participant satisfaction, in order to establish the feasibility and acceptability of the web-based intervention toolkit and questionnaires. Besides eligibility, baseline and post-program questionnaires, participants were interviewed about their overall experience with questionnaire filling and toolkit (Smailes et al., 2016). Additionally, toolkit usage analytics were obtained. The pilot feasibility study was conducted in order to better inform the final program and procedures. This study, as well as implications for final protocol are briefly described here.

#### Pilot study participants

Pilot participants were recruited among the running community through online advertisement. Study procedures were equivalent to those defined for the RCT, except that participants were not randomized, following the path of the study as if they were included in the intervention group. Participants were informed about the results of this pilot by individual reports.

The pilot feasibility study was a 6-week non-controlled trial without the follow-up. Considering an expected effect size of 0.23, 55 subjects per arm should be required in the main trial to detect significant differences at *p *= 0.05 (see sample size calculations for the main trial). Bearing in mind the rule of thumb for pilot sample size (Bell, Whitehead, & Julious, 2018), for an 80% powered main trial, with an expected medium effect size, the pilot should have between 10 and 15 subjects per arm.

#### Results of the pilot feasibility study

Due to recruitment problems, the eligibility criteria for participants with more than three months of running experience were more flexible. All other recruitment procedures were equivalent to the RCT.

#### Pilot participant characteristics

**Figure 5.**
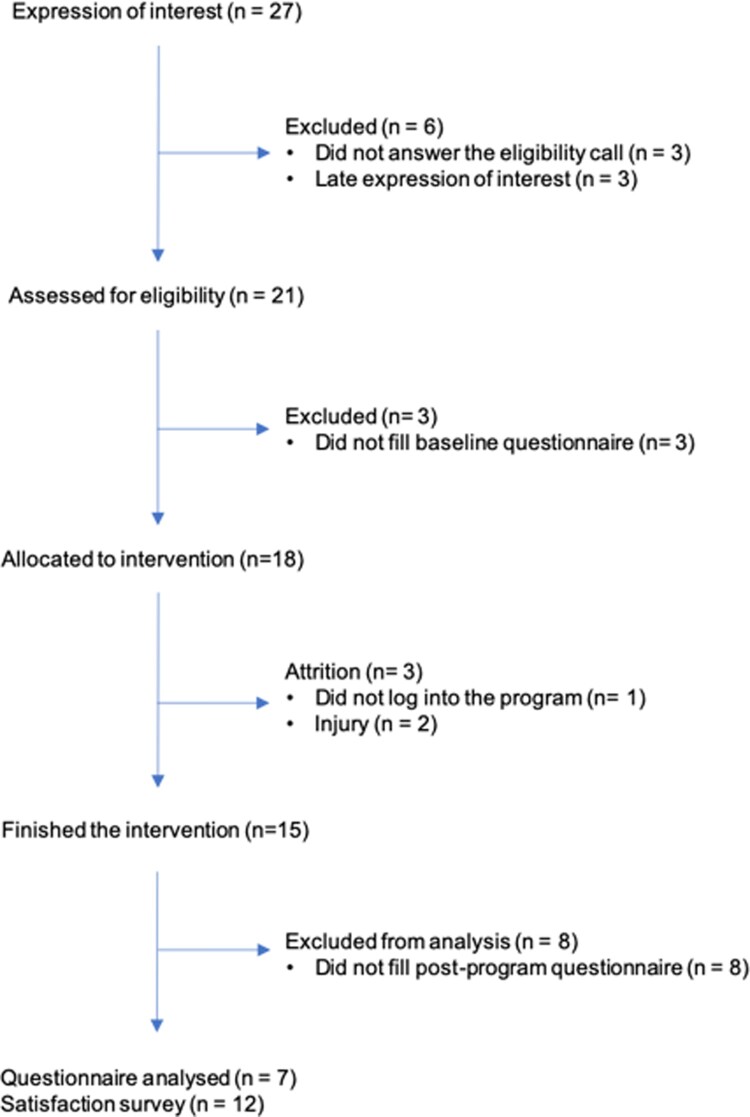
CONSORT diagram.

The CONSORT diagram for the pilot is shown in Figure 5. In total, 18 participants were enrolled and completed the baseline assessment. Of those, 3 dropped out during the intervention, allegedly due to time constraints and injuries; 15 participants finished the 6 week-intervention, 7 the post-program questionnaire, and 14 the overall study satisfaction questionnaire.

Participants (*N* = 18, 33% female), between 25 and 54 years-old (average 42.1 yrs.), were generally self-perceived as healthy (94%), highly educated (78%), and lived mostly in the Lisbon area (83%). Participants reported to run an average of 27 km and 4.4 h per week. Running experience ranged from 1 month to 30 years (average 6.3 yrs.). Most participants (88.9%) wore a running monitoring device to keep track of their running, social sharing and coaching, and 94.4% included other types of exercise in their training regime, such as warm-up and stretching, weight training or calisthenics, exercise classes or biking. A total of 61.1% used social media for motivation, education, interaction and coaching purposes.

#### Pilot adherence and program usage

From the 18 participants allocated to the intervention, 17 (94.4%) logged at least once, 50% logged once, and 22.2% twice. Only 6 (33.3%) completed the first session. Participants’ session completion decreased at sessions two (*N *= 5; 27.8%), five (*N *= 4; 22.2%), and only three participants (16.7%) completed sessions from eight to eleven. During the course of the program 12 participants (66.7%) logged at least one race (1–15 races, average 5.2), and the average run lengthened 9.4 Km (4–14 Km).

#### Pilot program evaluation: participant satisfaction

Overall, participants understood the purpose of the study, the concepts underlying the questionnaires and the toolkit, and felt supported by the research team. Most participants (83.3%) found the questionnaire too long. Participants generally found the toolkit website to be attractive and user friendly, classifying their overall experience with the study as positive. Most of the interviewed participants already used a running monitoring app, so they found the program to be less intuitive, repetitive in some questions and missing out important aspects such as ‘time of day, speed/ pace, and altimetry’. Additionally, participants reported problems with platform adjustment that led to ‘information overlap when used on cellphone’, the fact that ‘it didn't keep data inserted in previous sessions’ and ‘errors in google docs that forced them to restart the questionnaire’.

Three participants suffered injuries during the intervention period (25%). Two had muscular running-related injuries in the lower limbs and one had a foot trauma injury while playing football.

This pilot study had limitations that need to be considered, Due to recruitment constraints, the final sample included long time runners, which may differ from beginners, targeted by the RCT protocol. The low sample size and the absence of follow-up hindered further interpretation about the efficacy of the toolkit as it is.

## Ethics and dissemination

The study protocol was approved by the Ethics Committee of the Faculty of Human Kinetics, University of Lisbon (CEFMH 3/2018) and will be conducted in accordance with the declaration of Helsinki for human studies (World Medical Association, 2013). All participants will be informed about the possible risks of the investigation before giving their written informed consent to participate. Data from google forms (questionnaires), the intervention quizzes and toolkit analytics will be handled and stored in excel and SPSS files in a secure faculty server for ten years. Data will then be destroyed. All personal data will be anonymised at source. There will be no names or other identifiers in the manuscripts and qualitative descriptions whatsoever. Data will only be linked back to the individual via a separately stored coding system. Interventions will be discontinued if they are reported detrimental. Protocol deviations, violations and serious adverse events will be recorded by trial staff and monitored by the principal investigator (PJT) (MRC, 2017).

## Discussion

This paper presents the rationale, development, study design, methods and pilot application of the KOR trial, aimed at testing the effect of the intervention on the maintenance of recreational running behavior over time, after being voluntarily initiated by community-dwelling adults in the previous 3 months. This ‘light-touch’ intervention is unique in that it approaches running behavior and its sustainability, delivered in a digital format, and based on strong theoretical foundations.

We hypothesize that the sustainability of running behaviors will be higher in the SRM groups compared to the control group. Change in running behavior (e.g. frequency and minutes of running per week) and secondary outcomes related to the quality of the running experience (e.g. passion, flow, vitality) will be used as the dependent variables.

Additionally, the results of the pilot feasibility study, intended at testing intervention adherence, participant satisfaction, and questionnaire acceptance, were also presented. Overall, the pilot study indicated that the intervention has good acceptability, feedback was very positive, but toolkit interaction decreased after the first weeks, which is common in web-based interventions (Baumel & Yom-Tov, 2018). Moreover, the generalized use of other running-related devices, applications and communities may have overlapped with the anticipated content of the toolkit, challenging participants’ interaction. The completion ratio of the post-program questionnaire was low, maybe due to its extension and burden, but also because its timeline ended near the summer break.

The main trial should reduce questionnaire length and burden, and include interface with usual monitoring gadgets and apps (APIs), to increase data detail and prevent ‘unnecessary’ data insertion by the participants. Features such as feedback and social sharing, added to the goal setting features already integrated in the toolkit, may also increase attractiveness (Hosseinpour & Terlutter, 2019).

## Conclusion

This paper describes the rationale, the process of developing, study design, methods, and the pilot study of the KOR intervention. Valuable lessons are taken from the pilot feasibility study and adjustments will be made in the intervention and in its delivery, in order to improve adherence and overall experience. Results from the RCT will test the efficacy of this approach, contribute to the understanding of recreational running sustainability, and can be used in the development and optimization of future interventions aimed at physical activity promotion.
